# Effects of two types of exercise training on psychological well-being, sleep, quality of life and physical fitness in patients with high-grade glioma (WHO III and IV): study protocol for a randomized controlled trial

**DOI:** 10.1186/s40880-019-0390-8

**Published:** 2019-08-09

**Authors:** Dominik Cordier, Markus Gerber, Serge Brand

**Affiliations:** 1 Department of Neurosurgery, University Hospital Basel, 4031 Basel, Switzerland; 2 Division of Sport and Psychosocial Health, Department of Sport, Exercise and Health, University of Basel, 4052 Basel, Switzerland; 3 Center for Affective, Stress and Sleep Disorders (ZASS), Psychiatric Clinics (UPK), University Basel, 4002 Basel, Switzerland; 4 Department of Psychiatry, Substance Abuse Prevention Research Center, Health Institute, Kermanshah University of Medical Sciences (KUMS), Kermanshah, 6719851115 Iran; 5 Sleep Disorders Research Center, Department of Psychiatry, Kermanshah University of Medical Sciences (KUMS), Kermanshah, 6719851115 Iran

**Keywords:** Brain, Tumor, Glioma, Exercise, Training, Sleep, Control condition, Quality of life, Anxiety, Perceived stress

## Abstract

**Background:**

There is existing evidence on whether and to what degree regular exercise training improves the quality of life (QoL) among cancer survivors. However, in regards to patients with high-grade glioma (HGG; WHO grade III and IV), no conclusive study has been performed so far. The present trial aims to fill this gap by examining whether psychological well-being, sleep, QoL and physical fitness might be improved with two different types of exercise, as compared to an active control condition. Active control condition represent individuals participating at regular meetings to talk about their current life situation, though, the meetings were not intended as that of the psychotherapy group. Regular meetings are of the same frequency, duration, and intensity as the exercise interventions.

**Methods:**

A total of 45 patients with HGG after undergoing neurosurgery and adjuvant radiotherapy, chemotherapy, or chemoradiotherapy will be consecutively and randomly assigned to (a) an endurance training, (b) a resistance training or (c) to an active control condition. The intervention will last for 6 consecutive weeks, consisting of 2 weekly sessions (30–45 min per session). Measurements would take place at three time points, namely at the beginning of the study (baseline), 3 weeks after the beginning of the study, and 6 weeks after the beginning of the study. The last measurement also represents the end of the study. Aerobic exercise performance will be assessed objectively with a 6-min walking test, and a handgrip test will be used to assess the upper body strength. Further, participants will complete a battery of questionnaires covering sociodemographic information, QoL, sleep quality and sleep patterns, coping with stress, state- and trait-anxiety, depression, and fatigue. In parallel, experts will use the Hamilton Depression Rating Scale to determine and rate participants’ symptoms of depression.

**Significance:**

The present study will be the first to investigate and compare the impact of two different exercise modalities, namely endurance and resistance training, on physical fitness and dimensions of well-being, and sleep among patients with HGG who underwent neurosurgery followed by adjuvant radiotherapy, chemotherapy, or chemoradiotherapy. Importantly, unlike the majority of previous studies, the control condition consists of an active set-up to detect possible factual beneficial effects of exercise training, irrespective of social interactions.

*Trial registration* https://register.clinicaltrials.gov; identifier: NCT03775369

## Background

There is increasing evidence that cancer survivors benefit from adjuvant exercise training to improve their subjective wellbeing, and improvements have been observed in regards to quality of life (QoL), coping with stress, and self-control. Here, self-control refers to the individual’s ability to control her/his thoughts, emotions, and behavior. Exercise training also has the potential to counteract series of side-effects of chemotherapy or hormone therapy such as fatigue, weight gain, muscle loss, hot flushes, nausea, or increased susceptibility to infections [1, 2]. Further, Segal et al. [3] underlined in their systematic review that exercise training is a safe intervention, which provides benefits with regard to quality of life, and muscular and aerobic performance both during and after cancer-specific treatment. However, a deeper inspection of the type of cancer reported in the systematic review of Segal et al. [3] revealed that randomized clinical trials were performed for breast cancer, colorectal cancer, prostate cancer, or just mentioned ‘cancer’ without further specifications on the location, while no randomized clinical trial has yet tested the influence of exercise interventions in patients with glioma, and more specifically with high grade glioma (HGG; WHO grade III and IV). Thus, the present randomized clinical trial was designed to address this research gap, and to create an empirical evidence base on whether exercise training can be seen as a treatment option among patients with HGG during the post-surgery state in order to improve their wellbeing, sleep and QoL.

As mentioned by Ostrom et al. [4, 5] gliomas represent 31% of all malignant brain tumors and 81% of central nervous system (CNS) tumors diagnosed in the United States. Although gliomas are rare, they can still lead to significant mortality and morbidity. Further, not all types of gliomas develop in an aggressive way, and the heterogeneity of these gliomas in terms of histopathological types and grades, clinical outcomes, and genomics makes treatment and risk management even more difficult [6].

As regards to the identification of possible risk factors, Ostrom et al. [5, 6] summarized in their systematic review that ionizing radiation and heritable genetic variants appeared to increase the risk for gliomas, whereas allergies and atopic diseases appeared to be protective. However, there is inconclusive evidence of non-ionizing radiation via cell phone use and risk of gliomas.

The usual treatment algorithm for high-grade glioma consists of neurosurgical resection of tumor tissue and postoperative combined radiochemotherapy. Depending on the genetic sub-classification of the glioma and patient-specific factors such as age and clinical condition, postoperative treatment is sometimes given in the form of only radiation therapy or chemotherapy. In the present study, however, we will investigate whether and to what degree structured exercise training may have a (beneficial) effect on the psychological wellbeing, sleep, QoL, and physical fitness during the phase of postoperative adjuvant treatment.

The underlying rationales and research findings are as follows: On a physiological level, some scholars emphasize that regular exercise training has the potential to increase the levels of serotonin, brain-derived neurotrophic factor (BDNF) and endothelial growth factor (VEGF) [7], and to increase mitochondrial activity [8]. On the psychological level, other scholars argue that exercise training has the potential to improve the patients’ mood [9, 10] and self-esteem [11], to decrease symptoms of depression [12–17], anxiety, chronic pain [18], and above all rumination [19]. Further, in a previous study on the post-treatment of patients with post-aneurysmal subarachnoid hemorrhage, we have shown that compared to a control group, a 12-week regular endurance and resistance training ameliorated the symptoms of depression, insomnia and cognitive capacities [20–22]. Next, as regards to cancer survivors, most studies focused on breast cancer survivors during or after treatment [1]. Segal et al. [23] further emphasized that physical activity and exercise interventions were safe, provided benefit in quality of life, and might have the potential to enhance social exchange. In this respect, Brand et al. [19] showed that a single session of exercise could increase interest in social contact and interactions, at least among their investigated 129 inpatients (mean age: 38.16 years; median: 36.00 years; age range: 19–63 years) with a broad variety of psychiatric disorders. However, in regards to patients with HGG, to the best of our knowledge, there has been no study to investigate the impact of exercising on the patients’ well-being and QoL, despite it is well-acknowledged that individuals with HGG who underwent radiotherapy, chemotherapy, or both radio- and chemotherapy suffer from a dramatically decreased in QoL. Accordingly, the primary aim of the present study is to examine and compare the effects of two different types of exercise training (endurance vs. resistance) on psychological wellbeing (i.e., depression, stress, anxiety), sleep, QoL and physical fitness in patients with HGG who had neurosurgery followed by radiotherapy, chemotherapy, or both, compared to an active control condition.

## Methods

### Trial design

**Fig. 1:**
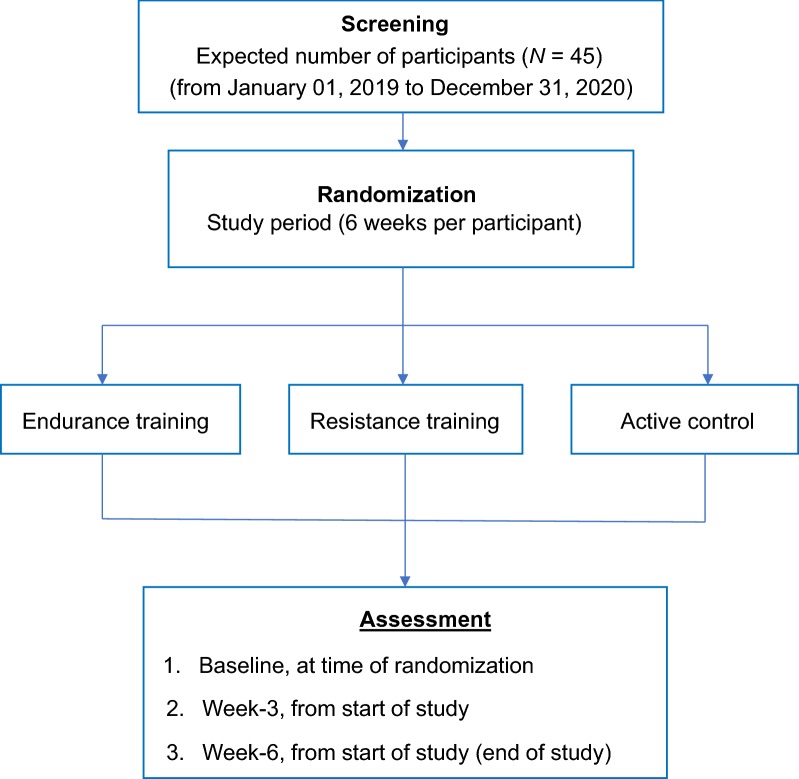
Study design. The enrolled participants are to be randomly assigned to one of the three study conditions: endurance training; resistance training; active control, with assessments to be taken at three pre-specified time points

The present study is an interventional, and randomized clinical trial. Each patient will participate for 6 weeks, with pre-specified assessment time points (Table 1): Measurements would take place at three time points, namely, baseline, which is the start of the study after the randomization. The second time point is 3 weeks after the baseline/beginning of the study. The third and last time point is 6 weeks after the baseline/beginning of the study, which also represents the end of the study. After written informed consent is obtained, eligible participants from the Basel University Hospital (Department of Neurosurgery; USB, Basel, Switzerland) are to be randomly and consecutively assigned to one of the following study grouped-conditions (Fig. 1): (a) endurance training, (b) resistance training, and (c) active control.

**Table 1:** Flow chart of the study: time points and the assessed dimensions at the time points

Outcome dimensions and time points	Time points				
	− 4 to − 1 weeks^a^	− 1 week	Week 0	Week 3	Week 6
Diagnosis and surgery	X				
Chemotherapy and/or radiotherapy at the same time			X X		
Patient’s eligibility is checked		X			
Signed written informed consent					
Randomization		X			
Assessment					
Cardiovascular performance (6MWT) and grip strength			X	X	X
Psychological functioning			X	X	X
Start study interventions			X		
Interventions			X X		
Study end					X

*6MWT* 6-min walking test, *X* this dimension is assessed; blue arrow, the time point from one period to the next

^a^− 4 to − 1 weeks, 4 weeks to 1 week of pre-treatment period

### Study population

Patients who meet the following inclusion and none of the exclusion criteria would be eligible for this study and consecutively enrolled in the study.

#### Inclusion criteria

1. Patients with high-grade glioma (grading criteria, WHO III and IV) after neurosurgical tumor resection or biopsy;
2. Had either postoperative chemotherapy, or radiotherapy, or both at the same time;
3. Age between 18 and 75 years;
4. Willing and able to follow the study intervention, and to comply with the study conditions;
5. Provide signed written informed consent;

#### Exclusion criteria

1. Severe psychiatric (psychosis, suicidal behavior, substance abuse disorder) issues;
2. Severe and somatic comorbidities (severe cardiovascular disease, severe diabetes, impairments of the musculoskeletal system); severe cardiopulmonary conditions;
3. Withdrawal from the study; either the patient wants to withdraw, or one of the principal investigators determines that due to unfavorable somatic or mental issues the participant is unsuitable for further participation in the study.

### Recruitment

The neurosurgeon (DC; 18 years of experience) is responsible for the recruitment of eligible participants.

### Participant timeline

As shown in Table 1, patients from the Department of Neurosurgery of the Basel University Hospital (Basel, Switzerland) have the following timeline: After neurosurgical treatment of the HGG, the patients would undergo either chemotherapy, or radiotherapy, or both. In parallel, they would be informed about the study content and the study aims. Once the written informed consent is signed, and the baseline assessment (cardiovascular performance and grip strengths; psychological assessments) (Table 1) is completed, the patients will be randomly assigned to one of the three study conditions. For the next 6 consecutive weeks, she/he would follow the schedule with two sessions per week. After the 3rd and 6th weeks, from the start of the study, the patients would be reassessed; similar as to the baseline assessments.

### Sample size calculation

We rely on two approaches: First, for pilot studies and clinical trials, Julious [24] recommended to recruit at least 12 participants/group. Second, we performed a power analysis with G*Power [25] with the following statistical preconditions: Cohen’s f for ANOVAs with repeated measures and within and between interactions: 0.25; alpha: 0.05; Power: 0.8; number of groups: 3, number of measurements: 3; calculated total sample size: 36. To counterbalance possible drop-outs, the sample size was set at 45 participants; 15 participants per group.

### Randomization

The randomizations are to be performed using an online software from the website Randomization.com (http://www.randomization.com). The online generator randomizes each subject to a single treatment using a method of randomly permuted blocks, with random block sizes for sex. Based on the generated list, a psychologist, not involved in the study, prepares 45 sealed envelopes, with no further identification, to be kept in an opaque and closed ballot box which are to be stirred before picking. Next, the psychologist would draw an envelope from the ballot and assign the study participants to one of the three study conditions picked. Once an envelope is drawn, it would be put aside. Experts rating the participants’ depression severity would be blinded to the participants’ study condition assigned.

### Interventions

#### Endurance training

The endurance training will last for 6 consecutive weeks and consists of 2 weekly sessions (30–45 min each). Sessions would take place in small groups of 2 to 4 participants. After 5 min of warming-up and stretching, participants will exercise for 25–35 min on the treadmill at a pace of 11 to 14 points on the Borg Scale of ranging from 0 (complete rest or resting pace) to 20 (maximum pace), followed by 5 min of cooling down. In this study, cooling down represents light exercise to allow the body to gradually transition to a resting or near-resting state. Participants are allowed to adapt to their pace and to take breaks throughout the session, though they are also encouraged to keep both their speed and pace as long as possible.

#### Resistance training

The resistance training will also last for 6 weeks with 2 weekly training sessions (30–45 min each), in small groups of 2 to 4 participants. After 5 min of warming-up, participants will follow a structured and standardized resistance training protocol to strengthen all main bodily skeletal muscles (upper and lower arms; shoulders, upper and lower legs; abdominal muscles; core muscles). The protocol is as follows: The participants would perform each exercise in 3–5 series of 10–15 repetitions per series, at 11 to 15 points on the Borg Scale, followed by the cooling down period. Intervals between the series of 10–15 repetitions will last for about 1–3 min.

#### Active control condition

For 6 consecutive weeks, participants of the control condition will meet twice per week for 30–45 min but by contrast to the endurance and resistance training condition, the participants would not perform any planned physical exercise. Each session would take place in small groups of 2 to 4 participants. The control condition is not a ‘bona fide’ condition, which would be actually intended to elicit change [26, 27], that is to say, by default, group sessions in psychotherapy follow the aim to change participants’ dysfunctional cognitive-emotional concepts and information processing; By contrast, in the present control condition, topics such as successful coping strategies are not treated and not proactively proposed by the clinical psychologist responsible to monitor the content of the control conditions. Rather, the participants would be encouraged to proposing and exchanging daily life experiences.

### Measures

#### Sociodemographic information

At baseline, the participants’ sex, age, civil status, highest educational level, and current job position will be assessed via a self-report.

#### Clinical- and glioma-related information

At baseline, clinical- and glioma-related information such as relevant past medical history, symptoms at initial presentation, neurosurgical procedure, histopathological diagnosis, and genetic subtype of the tumor would be retrieved from the participants’ medical records.

#### Psychological wellbeing

A set of self-report questionnaires will be used to assess the patients’ psychological wellbeing, including symptoms of depression, state- and trait-anxiety, and perceived stress. All questionnaires will be completed at baseline, 3 weeks into the study, and at the end of the study.

#### Depression

As in previous studies [28, 29], participants will complete the Beck Depression Inventory [30] to self-report symptoms of depression. The questionnaire consists of 21 items regarding typical dimensions of depression such as depressive mood, loss of appetite, sleep disorders, suicidality and similar. Answers are given on 4-point-Likert scales with the anchor points 0 (‘as always’; ‘no change’) to 3 (‘not able anymore’; ‘dramatic change’), with higher scores reflecting an increased severity of depressive symptoms. Evidence for the validity of the BDI has been previously reported [30].

#### State and trait anxiety

To assess state-and trait-anxiety, the State–Trait Anxiety Inventory (STAI) [31, 32] will be employed. The STAI consists of 42 items. Typical items for an anxiety state are “I feel relaxed”; “I feel nervous”, or “I feel tensed”. Typical items for anxiety traits are “I get nervous and restless when thinking at all my duties and issues”; “I can’t stop ruminating about unimportant stuff”. Answers would be given on an 8-points rating scale with the anchor points 0 (not true at all) to 7 (completely true), and with higher sum scores reflecting higher state and trait of anxiety. The STAI has been previously proved to have good psychometric properties in previous studies [31, 32].

#### Perceived stress

As previously described [33], we will employ the Perceived Stress Scale (PSS) [34], which has been proved to be a valid instrument in previous research [35], to assess subjectively perceived stress. The questionnaire consists of 10 items and is used to determine perceived overall stress occurring over the previous month. Answers are to be given on five-point rating scales ranging from 1 (never) to 5 (very often), with higher scores reflecting greater perceived stress.

#### Sleep

To assess subjective sleep complaints, participants will complete the Insomnia Severity Index [36, 37], comprising of seven items, answering on a 5-point rating scales (0, not at all, 4, very much), referring to the difficulty in falling asleep, difficulties in maintaining sleep, increased daytime sleepiness, and worriedness about sleep. The higher the overall score, the greater the respondent is assumed to suffer from insomnia. The validity of the ISI has been previously described [36, 37].

#### Quality of life (QoL, SF-36)

As previously described [38, 39], the short form 36 health survey (SF-36) [40] will be employed to assess the participants’ health-related QoL. This self-rating questionnaire consists of eight scales to assess impairments in physical activities (i.e., bathing, dressing, grocery shopping, using stairs, and using escalators) due to health problems (physical functioning), impairments in routine role activities at work or in other daily activities due to physical health problems (role limitations due to physical health), bodily pain, general health perceptions, energy and fatigue (vitality), impairments in social activities due to physical or emotional problems (social functioning), impairments in daily role activities at work or other everyday activities due to emotional problems (role limitations due to emotional problems), and general mental health. Answers would be given on different scales with different anchor points; interim scores would be transformed to achieve a range between 0 (worst QoL) and 100 (best QoL); thus, higher scores reflect a higher self-perceived quality of life. The validity of the SF-36 has been documented in previous research [41].

#### Physical fitness

To assess physical fitness, all participants will perform the 6-min walking test (6MWT) and handgrip strength test at baseline and at the end of the study.

We will use the 6MWT as a measure of cardiorespiratory fitness [42]. The 6MWT is a standardized, self-paced, submaximal exercise test, and has been designed especially for patients suffering from chronic diseases. Evidence of the validity of the 6MWT has been previously shown in various disease types [43–45] including cancer patients [46, 47]. The walking distance will be used as the main outcome measure of the 6MWT. Oxygen saturation before and after the 6MWT will be also taken into consideration.

As a measure of upper body strength, we will perform the grip strength test. As described elsewhere [48], maximum isometric grip force of the dominant hand would be assessed using a mechanic hand dynamometer (Jamar Handgrip Dynamometer; retailer: Rehaforum MEDICAL GmbH; Elmshorn, Germany). Participants will have three attempts per hand. The validity of the grip strength test has been previously documented, and researchers have shown that grip strength is associated with psychological wellbeing across different populations [49].

### Statistical analyses

Irrespective of which study conditions the patients are grouped to, at baseline, we will use Pearson’s correlations and univariate ANOVAs to examine the associations between the participants’ social and demographic background and their psychological wellbeing, sleep, QoL, and physical fitness. To examine whether and to what degree the three study conditions (endurance training, resistance training, active control) would impact the outcome variables over time, a series of ANOVAs for repeated measures with the following factors and dependent variables will be performed, namely, time (baseline; week-3 and week 6 since start of study); group (endurance training; resistance training; active control), and the time-by-group interaction; dependent variables: depressive symptoms, anxiety, stress, sleep complaints, QoL, cardiorespiratory fitness, and grip strength. Given the explorative character of the present study, we follow the previously reported recommendations [50, 51] and no correction of p-values for multiple testing would be performed. Data would be analyzed both per protocol and by intent-to-treat (ITT), with the last observation carried forward (LOCF). Patients will not be further followed up as regards the study questions, though, they would undergo routine checks every 6 months to monitor the development and recovery process of the HGG. The level of significance is set at alpha ≤ 0.05. All computations would be performed with the Statistical Package for the Social Sciences (SPSS)^®^ software (IBM Corporation, Armonk NY, USA) for Macintosh^®^.

### Ethical aspects

The study has been approved by the Ethikkommission Nordwest- und Zentralschweiz (EKNZ) (Switzerland; ProjectID 2018-01314), and the study is to be performed in accordance with the ethical principles laid down in the seventh and current edition (2013) of the Declaration of Helsinki. All eligible participants would be fully informed about the aims of the study and the confidential data handling. Although the interventions are not potentially risky, an insurance would be provided upon eligibility for participation. The insurance covers all damages and injuries occurring during the time a person is at the hospital to participate in the study.

### Protocol amendments

Important protocol modifications (i.e., change of assessment tools, inclusion, and exclusion criteria; change of interventions) would be communicated to the ethical committee (Ethikkommission Nordwest- und Zentralschweiz (EKNZ).

### Confidentiality and data protection

Participants would be fully informed and assured that their data are to be handled with highest confidentiality from the very beginning of the study, throughout the study and also afterward, once the study has been completed. Accordingly, no data and information would be shared with third parties. Staff members involved in the study would strictly comply with professional confidentiality. In this line, also members of the EKNZ ethical committee would be strictly obliged to respect medical confidentiality, and to strictly avoid any kind of divulgation of participants’ participation and data.

Only staff members of the present study are allowed to have access to the data and information of the study and participants. However, by law, members of the local ethical committee and members of the Swiss ethics committees on research involving humans are allowed to organize inspections and to get access to the source data. However, again, members of both authorities would strictly comply with professional confidentiality.

Data would be kept in an electronic database. Once a patient is enrolled in the study, she/he receives a study number, and the study number would be only visible in the database, while the principal investigator (DC) not otherwise involved in patients’ assessments and randomization has the patient sheet (“key”) with both patient’s name and her/his study number. In all of the files (Case Report Forms, statistic files, etc.) only patient’s study number would be registered.

### Monitoring

The source data/documents are accessible to independent monitors, who are not part of the research group, and questions are answered during monitoring. Again, audits and inspections of the authorities mentioned above may be performed to ensure proper study conduct and data handling procedures, as thoroughly described in the International Council of Harmonisation-Good Clinical Practice (ICH-GCP) guidelines and regulatory requirements.

### Study harms

After every intervention/session, participants would be investigated for any pain felt or other/additional unpleasant and unexpected sensations, which can be plausibly associated with the study intervention. Adverse events would be listed and discussed if the participant is to be excluded from the study.

### Ancillary and post-trial care

All patients would be treated, monitored, and routinely assessed as regards the development and recovery process of the HGG in the study center of Neurosurgery of the Basel University Hospital (Basel, Switzerland).

### Dissemination policy

Upon request, participants would receive their personal profile of cardiovascular performance, grip strengths, and psychological functioning. Further, results would be presented at national and international congresses on oncology, psycho-oncology, neurosurgery, and sport sciences. The results would be published in open-access journals. Last, upon request, data might be shared with researchers, who are proven experts in this field.

## Discussion

While there is a growing body of randomized clinical trials (RCTs) on the beneficial influence of regular exercise training on QoL in cancer survivors, such research refers exclusively to cancer such as breast cancer, prostate cancer or colorectal cancer [1, 3, 23]. By contrast, no such study has investigated the possible positive effects in cancer survivors with HGG. The present study expands upon previous RCTs in that: (1) two different interventions (endurance and resistance training) are compared with each other, (2) the control condition is an active control condition to ensure and to partial-out that the physical activity intervention per se, and not ‘merely’ the regular social contact with experts of the study center, is responsible for possible improvements to be observed, and (3) in that sleep quality is assessed; specifically as regards subjective sleep, there is substantive research to show that physical activity favorably impacts on sleep quality [52–54]. In our opinion, and from a methodological point of view, we believe the following reasons for the latter two aspects are of utmost importance.

First, as regards to the control condition, in a previous study [52] we successfully introduced a thoroughly assessed control condition to sort out that the effect of a regular jogging intervention in the morning on sleep and psychological functioning was related to physical activity intervention, but not to the social context. Specifically, all participants did meet at the same time in the same area of the school campus, took shower at the same time and had identical breakfast at the same time; thus, the only difference between the jogging and the control group was the physical activity intervention per se. Likewise, to assess the influence of adjuvant mindfulness-based stress reduction (MBSR) [55], participants of the control condition had the same frequency, duration, and intensity of social contacts with experts of the study center, as those participants undergoing the MBSR intervention. As mentioned, the active control intervention was not intended as being a ‘bona fide’ condition, which would have been intended to elicit dysfunctional cognitive-emotional processes. By contrast, in one of the very first studies on the influence of regular physical activity on depression among a smaller sample of female individuals with major depressive disorders [56], the intervention condition consisted of regular physical activity as group intervention, while to the control condition, no further contacts with the experts of the study center of with other patients were offered. Accordingly, Pilu et al. [56] correctly suspected that with such a methodological set-up, it was difficult to evaluate, if the improvement was due to a non-specific therapeutic effect associated with taking part in a social activity. In this view, in a former study, we showed that a single bout of physical activity could increase the interest of social interactions among individuals with psychiatric issues [19].

Second, in regards to sleep, there is a host of studies showing that regular physical activity has the potential to improve subjective and objective sleep [22, 52–54, 57–64]. However, such results are missing so far for both cancer survivors in general, and for cancer survivors with HGG. Further, there are also extant results to show that restoring sleep is associated with a broad range of cognitive, emotional, and behavioral advantages [65–70]. Accordingly, it is conceivable that also in the present study, improvements in physical activity are associated with improvements in sleep and psychological functioning.

### Anticipated outcome and significance

With the present study, we intend to investigate the influence of two different types of exercising, endurance training, and resistance training, on cardiovascular fitness, handgrip strengths, and dimensions of psychological well-being and subjective sleep in patients with HGG, as compared to an active control condition. Accordingly, we expect that compared to the active control condition, improvements would be observed in the two exercising conditions. If so, this study would provide evidence for the implementation of regular exercising in the clinical treatment of HGG patients.

## Data Availability

Once the study is completed, data would be available upon request from qualified researchers in the field.

## Abbreviations

6MWT: 6-min walking test
ANOVA: analysis of variances
BDNF: brain-derived neurotrophic factor
CT: computer tomography
DC: Dominik Cordier
EKNZ: Ethikkommission Nordwest- und Zentralschweiz
HGG: high-grade glioma
ICH-GCP: International Council of Harmonisation-Good Clinical Practice
ITT: intent-to-treat
LOCF: last observation carried forward
MBSR: mindfulness-based stress reduction
MRI: magnet resonance imaging
N: number of participants (whole sample)
QoL: quality of life
RCT: randomized clinical trial
SF36: Quality of Life Questionnaire; short form (36 items)
SPSS: Statistical Package for Social Sciences
VEGF: vascular endothelial growth factor
WHO: World Health Organization
